# Editorial: Personalized medicine—Where do we stand regarding bench to bedside translation?

**DOI:** 10.3389/fmed.2023.1243896

**Published:** 2023-08-14

**Authors:** Hanumantha Rao Balaji Raghavendran, Govindasamy Kumaramanickavel, Takeshi Iwata

**Affiliations:** ^1^Biomaterials Laboratory, Faculty of Clinical Research, Sri Ramachandra Institute of Higher Education and Research, Chennai, India; ^2^Narayana Nethralaya, Narayana Health City, Bengaluru, Karnataka, India; ^3^Molecular and Cellular Biology Division, National Institute of Sensory Organs, National Hospital Organization Tokyo Medical Center, Tokyo, Japan

**Keywords:** personalized medicine, ChatGPT, osteoarthritis, lateral sclerosis (ALS), ulcer prophylaxis

Personalized medicine is a cutting-edge subspecialty of medicine. The American Cancer Society states that “Precision medicine is a way for healthcare professionals to offer and plan specific care for their patients, based on the specific genes, proteins, and other substances in a person's body” (1). The term “personalized medicine” (PM) first appeared in 1999 (2). This is also known as individualized medication or treatment. Personalized medicine has experienced substantial growth in recent years in the medical and related professions. The International Consortium for Personalized Medicine (ICPerMed) asserts that technology innovation, as well as the advancement of the biomedical, social, and economic sciences, are what propel PM. Consequently, a significant investment in research and innovation is needed for its implementation to be successful. It is suggested that the vision recognizes PM as a medical specialty that focuses on the distinctive qualities of each individual. This will improve the effectiveness of diagnostic, therapeutic, and preventive measures, increase their economic worth, and ensure that all citizens have access to them (3).

A Pubmed search for the phrase “Personalized Medicine” produced 139,221 results on June 20, 2023. The core components of PM include clinical data, all-omics, imaging, and patient-generated digital tools (2). Using these fundamental components, preclinical models based on predisposition are developed, enabling early illness identification at the individual level (4). Since 2015, the FDA has approved 25% of new drugs that target PM at the molecular level. A report from the Personalized Medicine Coalition on FDA approvals in 2022 shows significant improvements in diagnostics and innovative treatments. Among the illnesses for which medications were approved were beta thalassemia, hemophilia B, cerebral adrenoleukodystrophy, refractory multiple myeloma, and particular subtypes of non-muscle invasive bladder cancer. The authorization of six molecular entities for rare genetic disorders. Twelve sophisticated tumor diagnostic testing techniques for individualized cancer therapy have been approved. T-cell engagers for uveal melanoma to elicit an immune response have been developed, promising therapeutics for non-small cell lung cancer with KRAS mutation (5). Efforts are being made to include PM in preventative treatment for the entire community. In contrast to the traditional gold standard model, which solely takes into account family history of diseases, the new model also takes into account clinical factors, polygenic risk, and family history in addition to family history. As a result, the pre-symptomatic stage is used to identify people who are vulnerable to age-related lifestyle problems, such as breast cancer (6).

With contributors from several nations, this issue's current topic is “*Personalized Medicine–Where do we stand regarding Bench to Bedside translation?*” The first paper discusses Organoids from Mucinous Appendiceal Adenocarcinomas as High-Fidelity Models for Individual Therapy. An organoids model can maintain the traits of the patient's cancer, according to Liu et al.. The effects of various chemotherapy combinations on patient outcomes were examined and investigated. Organoid's pathological characteristics were in line with the original tumor in the organoid model when it came to the expression of proteins including CK20, CDX-2, STAB2, CD7, and PAX8.

The second study by Linhartová et al., titled “CYP2C19 Gene Profiling as a Tool for Personalized Stress Ulcer Prophylaxis With Proton Pump Inhibitors in Critically Ill Patients - Recommendations,” provides recommendations. The idea is to use proton pump inhibitors to avoid stress ulcers specifically for each critically ill patient, using CYtheP2C19 gene profiling. According to the authors, stress-related ulcer prevention is not advised. They have explained that CYP3A4 and CYP2C19 are important players in the breakdown of proton pump inhibitors at a lower level. According to the study, proton pump inhibitor-using critically ill patients may benefit from personalized stress ulcer prophylaxis using CYP2C19 gene profiling.

Panio et al. explore the diagnostic circulating miRNAs in sporadic ALS in the chapter Diagnostic Circulating miRNAs in Sporadic Amyotrophic Lateral Sclerosis. One of the most fatal motor neuron neurodegenerative diseases, ALS has a high risk of fatality and disability. The authors assert that several therapy approaches had either failed or weren't very successful. According to experts who conducted a meta-analysis on miRNAs from the perspective of personalized medicine, the assessment of the expression levels of the blood miR-193b/miR-4745-5p combination could be employed in clinical practice for the diagnosis of sporadic ALS.

In a report from southern Italy, a multigene panel was used to screen a cohort of patients with breast/ovarian cancer for germline predisposing mutations. The section titled, Multi-gene panel testing increases germline predisposing mutations' detection in a cohort of breast/ovarian cancer patients from Southern Italy, by Nunziato et al.. This study presents the findings of a cohort of 64 patients who were BRCA-negative but had positive individual and familial histories of breast cancer and other cancer types. A patient's genomic DNA sample was used to create an enriched DNA library, which was then subjected to NGS analysis. They have come to the conclusion that additional molecular testing, particularly in cases where there is a family history, could lead to more precise cancer care.

Saffery et al. describe the typical response of CD14++ and CD16- monocyte to knee synovial mediators as a significant target element to stop osteoarthritis from progressing in their article Typical response of CD14++ CD16 – monocyte to knee synovial derived mediators as a key target to overcome them onset and progression of osteoarthritis. The Venn diagram employed by the authors of this original study indicated 55 proteins in the trauma baseline control synovium (TBCS) and 119 characteristic proteins in OA synovium (OAS). In contrast to TBCS, leukocyte-mediated immunity was demonstrated to be associated with OAS using string protein network analysis, which outlined a specific relationship between the protein and gene ontology. Overall, the study's findings indicated that customized therapy may be able to take advantage of the distinctions between traditional and OAS monocytes' protein profiles as well as those cells' motility, activation, and commitment in response to mediators.

Diagnostic genomics and customized precision medicine in ophthalmic disorders are discussed in the chapter by Panikker et al., “*Advancing precision medicines for ocular disorders: Diagnostic genomics to tailored therapies*.” The complexity of the methods used to identify novel genes and variants connected to a range of ocular illnesses has been considered in this review. a brief summary of the several types of vectors and human clinical trials that could allow personalized ophthalmic therapy and the use of genomes to identify retinal illnesses.

The method used in cutting-edge research is described in the Sun et al. article titled, *Digital Twin in Medicine: a Key to the Future of Healthcare*. The DT is a modern technological concept that links the physical and digital worlds. This technology makes it feasible to provide a precise diagnostic and individualized treatment. Digital twins may eventually attract more attention and turn into an essential instrument for tracking individualized health and access to personalized care, despite the fact that experts have pointed to data collecting, data fusion, and accurate simulation as limits. A large population could benefit from precise diagnosis and individualized care thanks to the development of high-resolution patient models made possible by big data and artificial intelligence.

Clinical studies are comparable to organ-on-a-chip and co-clinical investigations employing genetically altered mice (patient tissue, 3D cell cultures, and organoids) (4). In PM, cancer organoids created from patient tissue are investigated for therapeutic efficacy, pharmacological targeting, and medication customization (4). These methods advance clinical trial design while also advancing our understanding of the causes of disease.

PM has a bright future, according to the ChatGPT, or Generated Pretrained Transformer conversation (7). Although ambitious, the PM era, which is only getting begun, holds the key to a paradigm shift in the detection of illness propensities, the understanding of disease mechanisms, the development of novel molecular drugs, and the efficient design of clinical trials (8). Population-level implementation of PM in healthcare is extremely difficult due to the high cost of new technology and the complexity of the geographical and cultural settings. However, challenges will be met with opportunities and solutions.

In conclusion, it is anticipated that advances in genomic approaches to ocular disease diagnosis, personalized stress ulcer prophylaxis, organoid models for breast and ovarian cancer, amyotrophic lateral sclerosis, specific cd markers and monocyte migration in osteoarthritis and OA synovium, and finally the significance of digital twin technology for developing precise protocols will all contribute to the cutting-edge knowledge in the field of tailored or personalized medicine.

## Author contributions

HR and GK framed the concept and drafted the content. TI edited the final draft.
